# Telemedicine home-based management in patients with chronic heart failure and diabetes type II: study protocol for a randomized controlled trial

**DOI:** 10.1186/s13063-024-08171-0

**Published:** 2024-05-21

**Authors:** Palmira Bernocchi, Vittorio Giudici, Gabriella Borghi, Patrizia Bertolaia, Salvatore D’Isa, Roberto Trevisan, Simonetta Scalvini

**Affiliations:** 1 Continuity of Care Service, Istituti Clinici Scientifici Maugeri IRCCS, Institute of Lumezzane, Via G. Mazzini 129, 25065 Lumezzane, Brescia, Italy; 2 Department of Cardiac Rehabilitation, Bolognini Hospital, Azienda Socio-Sanitaria Territoriale Bergamo Est, Seriate, Bergamo, Italy; 3 Socio-Health Management Direction, Azienda Socio-Sanitaria Territoriale Bergamo Est, Seriate, Bergamo, Italy; 4 Cardiovascular Department, Cardiology Unit, ASST Papa Giovanni XXIII, Bergamo, Italy; 5 Endocrinology and Diabetes Unit, ASST Papa Giovanni XXIII, Bergamo, Italy; 6 Cardiac Rehabilitation Unit, Istituti Clinici Scientifici Maugeri IRCCS, Institute of Lumezzane, Brescia, Italy

**Keywords:** Heart failure, Diabetes mellitus type II, Tele-health, Telemedicine, Teleassistance, Telemonitoring, Telerehabilitation, 6-min walk test, Motivational feedback, Randomized controlled trial

## Abstract

**Background:**

Heart failure and type 2 diabetes are prevalent public health issues in Europe. These complex chronic conditions require extensive pharmacological management, ongoing self-care, and behavioral changes. Despite the known benefits of lifestyle changes, such as regular exercise and better control of blood sugar levels, patients may need help implementing the recommended changes. This study aims to assess the effectiveness of a telemedicine program for managing heart failure and type 2 diabetes at home. The program focuses on promoting lifestyle changes.

**Methods and analysis:**

During scheduled outpatient cardiology evaluations, eligible patients are recruited and randomly assigned to either an intervention or control group in a 1:1 ratio. The intervention group receives support from a nursing case manager through a structured home-based teleassistance program and a trainer for daily physical activity stimulation. They also have access to teleconsultations with cardiologists and diabetes specialists as needed, telemonitoring of vital signs, and daily step tracking. An app records and monitors daily drug treatment, glycemia, blood pressure, heart rate, and other clinical parameters. Patients can also self-report symptoms and communicate via a chat and videoconference system with a Nurse Case Manager. The control group receives routine care. Data collection occurs before intervention and 6 months after baseline during a new outpatient cardiology evaluation. The primary outcome is to measure the difference in the distance walked during a 6-min walk test between baseline and after 6 months. The key secondary outcomes include improving the disease status and physical activity profile. Data will be analyzed according to the intention-to-treat principles.

**Discussion:**

This study will provide evidence on the efficacy of a telemedicine home-based management model to maintain correct lifestyles in patients with both heart failure and type 2 diabetes, improving self-management, their empowerment on the diseases, and increasing their knowledge and ability to recognize symptoms early.

**Trial registration:**

ClinicalTrials.gov NCT05633784. Registered on November 30, 2022.

**Supplementary Information:**

The online version contains supplementary material available at 10.1186/s13063-024-08171-0.

## Background

As the population of industrialized countries ages, there is a significant rise in chronic diseases. Heart failure (HF) and type 2 diabetes (T2DM) are prevalent public health issues in Europe. HF affects 1–2% of adults and increases to over 10% in those aged 70 years or older [1, 2]. T2DM is continuously growing and is a particular concern in HF, where it is greater than 10% [3–6].

The weight of comorbidities is one of the main factors modifying the risk of hospitalization for HF [7, 8]. These complex chronic conditions require extensive pharmacological management, ongoing self-care, and behavioral changes (diet modification, sodium restriction, weight, and blood glucose monitoring). Adherence to recommendations regarding lifestyle modifications, such as increasing physical activity, is often limited despite the favorable effects these changes have on the chronically complex patient, as recommended by European and American guidelines [1, 2, 9, 10]. In patients with T2DM, more daily steps are associated with lower glycated hemoglobin (HbA1c) values and reduced waist circumference and body mass index [11, 12].

A complex drug regimen, typical of these diseases, is often associated with low adherence in patients with HF and T2DM, but it is an essential factor for improving health in patients with HF and DM [13]; on the contrary, patient adherence continues to be poor, leading to errors, adverse events, hospitalizations, and death [14, 15]. Therefore, interventions are needed to improve all these factors and optimize adherence.

Many programs try to change unhealthy lifestyles and help patients maintain a healthy life for as long as possible. In the Information and Communication Technology era, Telemedicine could be an alternative to promote healthy behaviors. A customized home telemedicine program founded on individual needs and risk profiles might be the best approach to plan the follow-up care of patients with complex chronic conditions. These programs should include routine self-management support, outcome measures, and electronic information systems to share patient data among the healthcare professionals engaged. These programs can provide tailored and scalable solutions for high-risk populations with multiple comorbidities, especially those with chronic illnesses, and promote continuity of care, allow for disease monitoring, prevent complications, and reduce disability [16, 17].

Patients with chronic and comorbid conditions require a comprehensive home care plan. Through our past experiences, we have found that telemedicine programs, which include teleassistance, telemonitoring, and teleconsultation, help these patients manage their conditions and take control of their health [18, 19].

Telemedicine can assist in recognizing symptoms early, managing medical devices and apps, identifying obstacles to adherence to therapy, such as adverse effects of drugs, checking if the intensity of physical therapy is adequate, and facilitating communication between professionals, such as nurses, physiotherapists, general practitioners, specialists, and pharmacists. This collaboration among healthcare providers is crucial for ensuring the best possible care for the patient and improving their overall awareness of their health status [18, 19]. Involving family members in the educational plan is essential for success and critical to the outpatient phase.

In this context, the nurse has a central role in improving the patient’s ability to increase self-management and empowerment and needs particular communication skills [20–22].

Furthermore, telemedicine applications, such as digital devices for recording and sending vital parameters and apps, can significantly change the quality of communication and the working methods of professionals [23].

Digital devices and mobile apps integrated into the treatment of chronic diseases can aid in the early diagnosis and treatment of potential disease instabilities. However, it is essential to consider how patients may react to the introduction of digital technology, as only some are technologically prepared. To ensure successful implementation, end-user guidance during the design and development is crucial [24].

The presence of a reference figure can assist patients in overcoming any apprehension they may have about using technology. Patients tend to be more receptive to technology when they believe it is crucial. All this can ultimately lead to a better patient experience and outcome [25].

The proposed trial (NET-2018–12367206-3) is part of the Italian Network Project “TELEMECHRON study” (NET-2018–12367206) [26] and intervenes to approach the solution of the medical-nursing and management problems that patients with chronic diseases face at home.

Our study aims to test the effectiveness and feasibility of an integrated, home-based medical/nursing intervention with trainer support for exercise versus conventional care in patients with CHF and T2DM. This paper aims to describe the trial’s design and rationale.

## Methods/design

### Study design and setting

This study (ClinicalTrials.gov ID: NCT05633784) is a consecutive, multicenter, open, randomized controlled trial. All patients are enrolled during the scheduled outpatient disease control visit (T0) and seen again in the outpatient department after 6 months (T6).

The study strategy is registered, constructed, and presented according to the recommendations for Interventional Trials (SPIRIT) [27] (SPIRIT checklist, Additional file 1) and Consolidated Standards of Reporting Trials (CONSORT) guidelines [28].

**Fig. 1:**
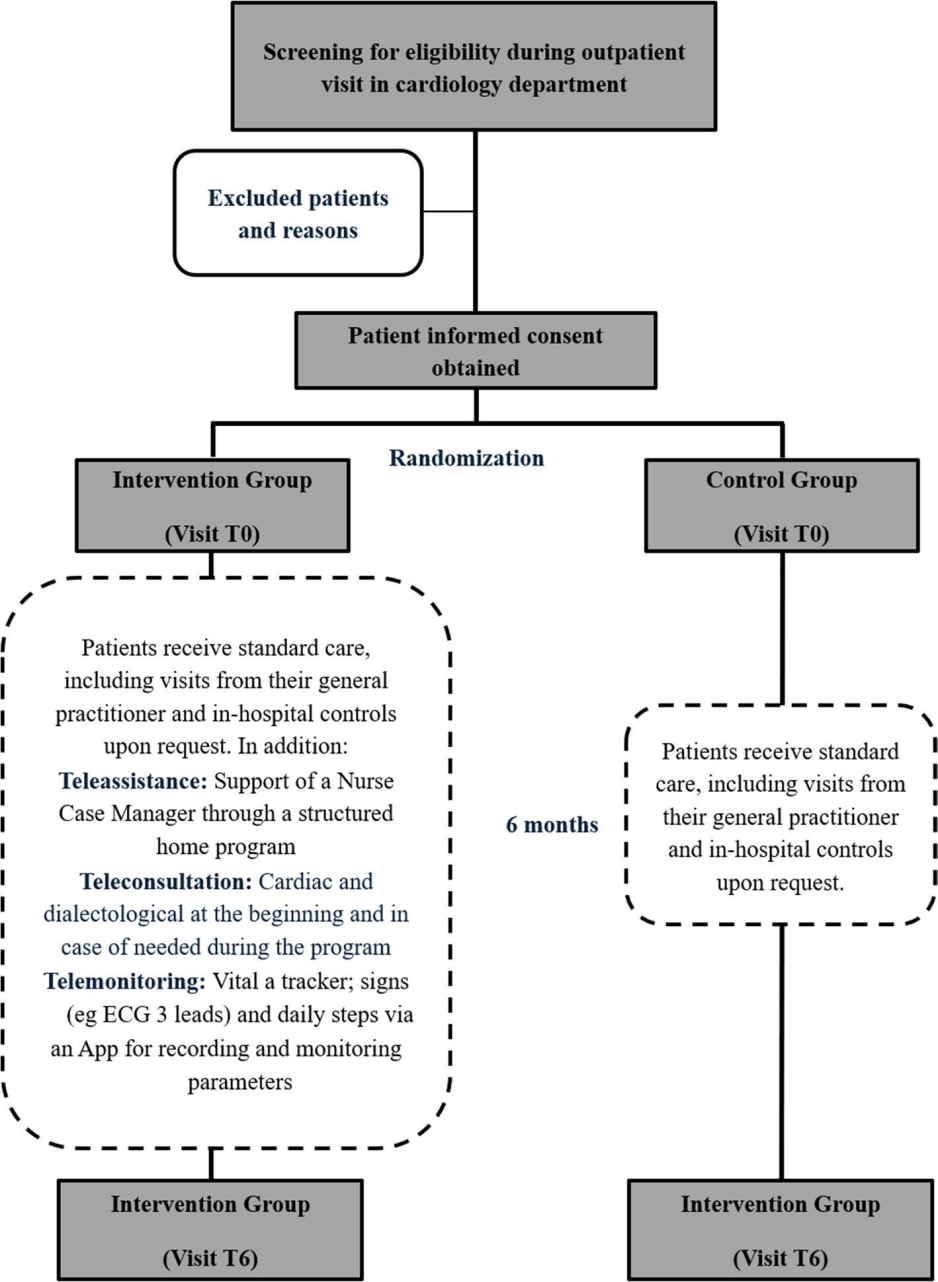
Flow chart of the study design

Figure 1 shows the flow chart of the study design.

### Study sites and patient population

Patients are recruited from the Cardiology Departments of three hospitals in the Lombardy Region, Italy (Istituti Clinici Scientifici Maugeri IRCCS, Institute of Lumezzane, Brescia; Bolognini Hospital, Seriate, Bergamo; Papa Giovanni XXIII Hospital, Bergamo).

### Eligibility criteria

The inclusion criteria are as follows:

- Patients aged ≥ 18 years
- A documented diagnosis of HF (HF with reduced or preserved ejection fraction) without hospitalization due to decompensated HF in the last 3 months
- Diagnosis of T2DM in treatment with antidiabetic drugs
- Ability to walk without aids
- Written informed consent
- Consent to the use of devices and app

The exclusion criteria are as follows:

- The new-onset HF diagnosis
- Life expectancy of less than 6 months
- Medical issues that preclude participation in the program
- Physical activity limitation
- Impossibility of using mobile technology

### Randomization

Patients are screened in the outpatient departments of the three hospitals during routine HF visits. A Nurse Case Manager reviews the scheduled office visits and personal electronic records weekly to identify patients eligible for the study. The study physician verifies their eligibility based on the inclusion criteria during the office visit and screens any other patients. All eligible patients are asked to participate, and those who give their consent are randomized (1:1) into either the intervention (IG) or control group (CG). Patients are allocated using a computer-generated table in fixed blocks of four, with the allocation sequence concealed from investigators to prevent selection bias. A separate randomization list is created for the three centers involved.

Due to the intervention’s nature, the patients and physicians know group allocation. However, outcome assessors and data analysts will be blinded to the allocation.

We have standardized the nursing approach as much as possible by conducting joint staff training, organizational meetings, and planning before patient enrolment in the three hospitals involved.

### Trial structure

At time T0, for patients randomized in the study, the following information is collected:

1. Demographic variables: age and gender
2. Clinical variables: ejection fraction, NYHA class, and comorbidities
3. The Cumulative Illness Rating Scale (CIRS) [29] measures the patient’s health status. Each item is evaluated according to an ordinal scale with increasing levels of severity: from 1 (absent pathology) to 5 (very severe pathology). Two measures are obtained:
  Severity Index (SI): the average of the scores of the first 13 categories (the maximum obtainable score is 5)
  Comorbidity Index (CI): the category numbers with equal or higher scores.

At times T0 and T6, in all patients randomized, the following information is collected:

1. Physical variables: body mass index (BMI), abdominal circumference
2. Biochemical parameters: glycated hemoglobin (HbA1c) and the amino-terminal fragment of Brain Natriuretic Peptide (NT pro-BNP).
3. Evaluation of exercise capacity by the 6-min walk test (6MWT) [30, 31].
4. The Physical Activity Scale for the Elderly (PASE) [32] assesses physical activity performed during the previous period.
5. Quality of life is measured in the following ways:
  The Minnesota Living Heart Failure Questionnaire (MLHFQ) [33]. MLHFQ comprises 21 topics that evaluate how, over the past month, the various physical and emotional symptoms of HF have prevented you from living as you would like.
  The Diabetes Quality of Life (DQOL) questionnaire [34]. DQOL comprises 46 questions that investigate four areas: general satisfaction, the global impact of the disease, concerns about social relationships, and concerns about diabetic disease. The scores on each scale range from 0 to 100; higher scores correspond to a better quality of life.
  The validated Italian version of the 12-item Short Form Survey (SF-12) questionnaire [35, 36]. The SF12 has a 6-item Physical Component Summary (PCS) and a 6-item Mental Component Summary (MCS).

Only in the patients of the IG are evaluated the weekly mean in the number of steps from baseline over the 6 months of follow-up, and at T6:

1. Satisfaction with care. Six items, with a score from 0 (not at all satisfied) to 4 (very satisfied), investigate the service as a whole, the use of the devices, the willingness of healthcare professionals to respond to the patient’s needs, the clarity of indications to the suggestions provided, the feeling of support, and whether the service is perceived as a real help or not (Table 1).
2. Usability of the devices and the app through the System Usability Scale (SUS) [37–39].

**Table 1:** Customer satisfaction

1. **How do you judge the system overall?**	
a. Not satisfying at all	0
b. Poorly satisfying	1
c. Fairly satisfying	2
d. Quite satisfying	3
e. Very satisfying	4
2.Was it easy to use devices?	
a. Very complicated	0
b. Quite complicated	1
c. Complicated	2
d. Quite easy	3
e. Very easy	4
3. **Did you experience difficulties in contacting the service?**	
a. Very frequently	0
b. Frequently	1
c. Sometimes	2
d. Rarely	3
e. Never	4
4. **Were the indications of the health staff clear?**	
a. Not at all	0
b. Poorly clear	1
c. Fairly clear	2
d. Quite clear	3
e. Very clear	4
5. **Do you feel more secure since when you have access to the service?**	
a. Not at all	0
b. Poorly	1
c. Fairly	2
d. Much	3
e. Very much	4
6. **Did the access to the service help your family or the people you live with?**	
a. Not at all	0
b. Poorly	1
c. Fairly	2
d. Much	3
e. Very much	4

**Fig. 2:**
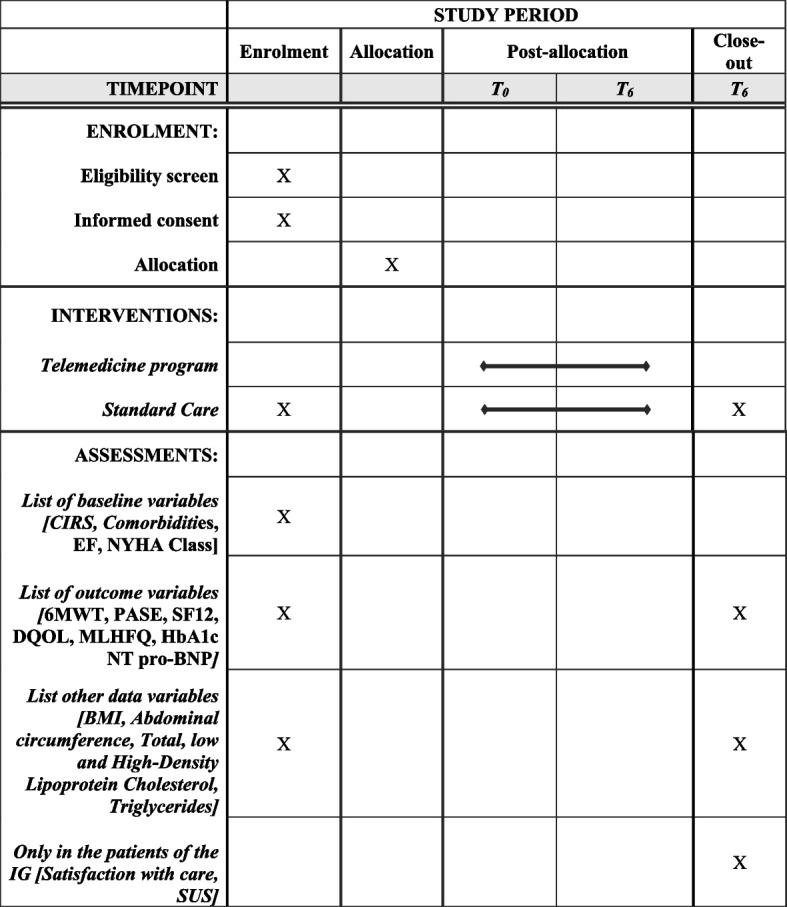
Complete treatment and assessment phases

The complete treatment and assessment phases are available in Fig. 2.

### Technology support

The protocol makes use of two main components:

- ✓ HealthPlatform web portal and app (CompuGroup Medical SE, CGM, Italy)
- ✓ TreC Cardio web portal and app (Fondazione Bruno Kessler, Trento, Italy)

And two wearable devices:

- ✓ Electrocardiograph (Hi – ECG 3 leads device, CompuGroup Medical SE, CGM, Italy)
- ✓ Tracker bracelet Fitbit Inspire 2 (https://www.fitbit.com/)

HealthPlatform is a software platform consisting of a web portal for patient management by healthcare personnel and an Android/iOS smartphone app used by patients for clinical data collection and communication with doctors and nurses. The HealthPlatform system is Medical Device Class IIa certified, and the channel between the app and the back-end is protected using the Transport Layer Security protocol (TLS with encryption).

The platform acquires:

1. Vital parameters through a Hi-ECG device with three leads transmit electrocardiogram traces to a smartphone via Bluetooth. The collected data are saved on the smartphone’s internal app database and associated with the user. The smartphone app is a gateway to recognize the associated ECG and sends the data to the server via the HealthPlatform web platform.
2. The fitness data recorded by the tracker automatically flows to the Fitbit App and the Fitbit server and will be retrieved daily from the HealthPlatform server. Data acquisition via Bluetooth is carried out directly by the Fitbit app, which sends them to the Fitbit server independently from the HealthPlatform app. The HealthPlatform server’s task is to interface directly with the Fitbit server and retrieve the data associated with the user daily (the integration is, therefore, server-to-server side).

The doctor/health personnel can access a specific section of the HealthPlatform web portal using personal credentials to view patients’ electrocardiographic traces and FitBit data.

The “TreC Cardio” platform includes a web dashboard for healthcare personnel to manage patients and an Android/iOS app for patients to collect clinical data and communicate with doctors and nurses. With the “TreC Cardio” app, patients can:

- View their medication history and upcoming doses
- Confirm whether they have taken their daily therapy or provide a justification for not taking it
- Enter, view, and modify self-detected clinical parameters and symptoms
- Receive reminders for healthcare actions (e.g., measuring blood pressure)
- Chat, send images/PDFs, and make video calls with healthcare personnel

Once authenticated, healthcare personnel in the web dashboard can:

- Enroll new patients and activate the TreC Cardio app for them
- Manage individual patient treatment plans by activating therapies prescribed by specialists and tasks/actions
- Remotely monitor patient data and view entered data trends
- Deliver questionnaires via chatbot
- Chat and conduct video calls with patients

**Fig. 3:**
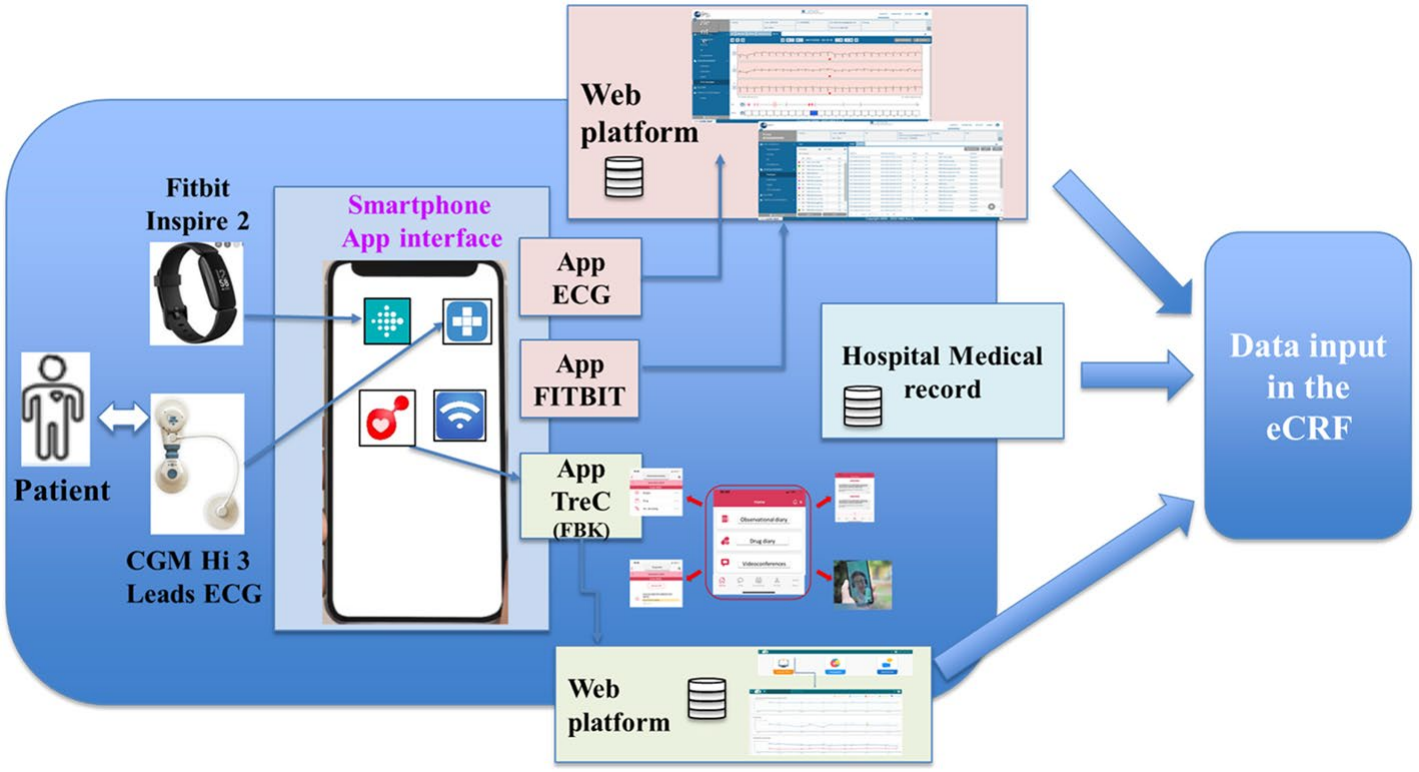
Technology model of the protocol

Figure 3 represents the technology model of the protocol.

### Interventions

#### Intervention group

Patients in the IG group are followed through a remote home telemedicine program for 6 months, in addition to standard care, such as visits to the general practitioner and hospital control on request.

The telemedicine program is characterized by the following:

1. Supporting from a nursing case manager through a structured teleassistance program (phone or video calls at least once a week)
2. Cardiology and diabetes teleconsultation at the beginning and in case needed during the program.
3. Support of the trainer to stimulate daily physical activity
4. Telemonitoring of patient vital signs (e.g., single electrocardiographic trace) and daily steps via a tracker
5. An app for recording and monitoring parameters: daily drug treatment and clinical parameters

#### Intervention program

Upon enrollment, the patients receive the tracker bracelet, the electrocardiograph device, and a smartphone with pre-installed apps for receiving the respective information on wearable devices.

The Nurse Case Manager coordinates activity and promotes patient learning of disease self-management techniques to prevent exacerbations; she/he plays a central role in all home care continuity interventions, becoming a key interface in the dialogue between patients and specialists.

The educational plan involves family members and is crucial to the program’s success.

An agenda of scheduled contacts is shared with the patient.

During the teleassistance program in the 6-month home-based study period, the nurse:

- Collects information regarding medical history and symptoms
- Provides health education and training
- Verifies prescription adherence
- Evaluates general well-being
- Assesses the level of assistance provided by the caregiver
- Updates the computerized clinical file
- Activates specialist intervention for a teleconsultation if necessary

Patients can contact their nurse during operating hours for clinical, symptom, or treatment-related issues.

Patients are encouraged to perform physical activity at least thrice a week, reinforcing the importance of lifestyle changes and exercise, asking them always to wear their Fitbit to monitor their daily steps constantly.

Furthermore, the patients are encouraged to update the “TreC Cardio” app information by entering any symptoms and biological parameters (blood pressure, heart rate, weight, blood sugar, etc.), viewable by the nurse on the web dashboard.

The healthcare personnel will be able to remotely manage the treatment path of the individual patients enrolled by prescribing therapies and tasks, view the progress of the clinical parameters collected and entered by the patient (e.g., vital signs, symptoms), interact with the patient through a chat for exchange of text messages and the sending of multimedia files by the patient, activate a videoconference, and deliver questionnaires.

The research team continuously monitors the data to ensure patients comply with the intervention. Patients are contacted if the nurse receives signs and symptoms of a worsening clinical condition to resolve problems with device malfunctions or in the event of failure to enter information into the app.

### Control group

After the baseline assessments required by the program, patients in the CG receive standard care, including visits from their general practitioner and in-hospital controls upon request. Patients are informed about the importance of maintaining a healthy lifestyle and encouraged to engage in daily physical activity. Information leaflets on disease management and physical activity are provided. Final evaluations will occur at the 6-month cardiological visit.

#### Outcome assessments

##### Primary outcome measures

The primary outcome is exercise tolerance improvement measured by the difference in the meters walked at the 6MWT. The test measures the distance walked when subjects are asked to walk as fast as possible for 6 min, and performance in this test has been used to measure cardiovascular exercise capacity, particularly in patients with congestive heart failure [30] and diabetic patients [31].

##### Secondary outcomes measures

1. The improvement of the physical activity profile by the PASE, a self-reported questionnaire of occupational, household, and leisure items over 1 week
2. Improvement of QoL
3. Improvement of the disease status (NT pro-BNP, HbA1c)
4. Reduction of hospitalizations for all causes

#### Withdrawal

According to Italian research ethics legislation, we inform the patients about their rights as subjects in a scientific trial and their discontinuation rights. We do this to make patients consider participation thoroughly to diminish the likelihood of dropping out. Patients can withdraw from the trial at their request or the request of their legal representative at any time. Every withdrawal is recorded in the “personal health record” of the patient.

#### Data collection, management, and analysis

We use RedCap, an eCRF web tool, to manage anonymized surveys and databases. Each hospital stores personally identifiable information and clinical variables; only authorized personnel can access the data. The project manager and nurse hold the code list connecting personal identifying data to the individual participant. The principal investigator from each hospital is responsible for securing and monitoring data collection and interpretation, as well as being involved in project management, analysis of samples, data collection, and observations, and they will jointly interpret the results.

All adverse events that occurred during the 6-month study observation period will be reported in the final paper. All serious and unexpected adverse events will be reported to the Ethics Committee as required.

#### Sample size

Based on the literature analyzed [40, 41] of the experience developed over the years in patients with heart failure [42] and patients with a complex phenotype, SCC and COPD [19], followed by remote surveillance, we estimated a sample of 240 patients who could be evaluated to demonstrate efficacy on the primary outcome. The number should be sufficient to establish an anticipated difference of 45–50 m in walking distance at the 6MWT (primary endpoint) between the intervention and control group to demonstrate a statistically significant difference between groups applying a *t*-test with alpha = 0.80 and beta = 0.05. A mean of 360 m (SD = 115) was considered. We performed a comparison test between two independent means (unpaired data) with equal sd (sd1 = sd2 = sd) in the two groups. Therefore, assuming an SD = 115 and delta = 28,217, the sample is 208 patients, 104 per group. Thus, considering the 15% of patients not present at T6 for calculating the endpoint (dropout), the sample becomes 239.

### Statistical analysis

Statistical analysis will be carried out by a certified health professional using STATA software (College Station, TX, USA). Data will be descriptively analyzed and presented as percentage or mean ± standard deviation for all clinical variables, median ± interquartile (IQ) range for variables without a normal distribution, and percentage for categorical and binary variables. The Kolmogorov–Smirnov test will test the distribution and normality of variables. To compare groups at T0, the Student *t*-test, the Mann–Whitney-Wilcoxon test for continuous variables, and the chi-squared test for categorical variables will be used.

Two-way analysis of variance (ANOVA) will analyze the effects of the intervention for repeated measures (time and group). A post hoc analysis will be conducted when the ANOVA *F* ratio is significant for the Student *t*-test among times and groups, and Bonferroni’s correction will be applied. Statistical significance is defined as *P* = 0.05.

Data will be primarily analyzed according to the intention-to-treat principle. An on-treatment analysis will be subsequently performed to assess whether protocol deviations have caused bias. Participants with documented deviation from the study protocol (i.e., patients in the intervention group who received only some of the intervention or participants in both groups with incomplete follow-up data) will be excluded.

No interim analysis is planned as no harm to patients is expected from this trial. There is no reason to stop prematurely other than the end of the financing.

### Oversight and monitoring

ICS Maugeri, as Project Management Group, composed of healthcare professionals (specialists, nurses, and therapists) and experts, coordinates the implementation of the study and the maintenance of the trial’s IT system and verifies that patient data collected anonymously into the RedCup platform is constantly inserted in the three centers involved in patient recruitment. The project Management Group will perform the final analysis of data. The steering committee comprises healthcare professionals (lead investigators) and experts from the three centers involved.

No groups of patients or the public are involved in this specific trial. The study, based on the experience acquired over the years, was designed considering the centrality of the patient and a personalized approach.

A Data Monitoring Committee is not involved as this protocol is a low-risk intervention.

To ensure data safety and comply with the General Data Protection Regulation (EU GDPR 2016/679) rules, a Data Protection Impact Assessment (DPIA) and security assessment were performed on all platforms, devices, and apps, with the assistance of a data security officer.

No auditing was planned for this trial.

The Ethics Committees will be informed of the progress and emerging issues.

Any changes to the protocol that may impact the conduct of the study will be submitted to the competent Ethics Committees, who will notify funders. A copy of the revised protocol will be sent to the PI to add to the Investigator Site File. Any deviations from the protocol will be fully documented using a breach report form, and we will update the protocol in the clinical trial registry.

### Dissemination

The study’s results will be disseminated through seminars, social media, and publication in a peer-reviewed journal to the Telemechron study group, project stakeholders, and healthcare professionals. Investigators will share authorship.

## Discussion

This study aims to test the effectiveness of a Home Telemedicine program to help patients suffering from both heart failure and type 2 diabetes maintain stable clinical conditions and improve correct lifestyles. The proposed program intervenes in the multiple clinical and management problems the patient with chronic pathology must face daily. The team that follows the patient at home coordinates and encourages the learning of self-management techniques for the disease, allowing the patient to identify sub-critical conditions preventing flare-ups as much as possible. The educational plan involving patients and, where possible, their families is crucial to the program’s success and constitutes a predominant component throughout the entire process. The nurse and the trainer take on a central role in all home care continuity interventions and become an essential interface in the dialogue between patient and specialist. The other significant challenge of this project is the introduction of a non-digital native-age population integrating digital devices and mobile apps in a treatment model for chronic diseases that can help in the early diagnosis of potential instabilities and improve treatment adherence. Many articles report that [43, 44], despite their apparent advantages, age remains a significant barrier to the acceptability of digital devices. In our program, the main components are teleassistance provided by a nurse as a case manager, the integration of telemonitoring of vital signs, and an app to manage and share multiple patient parameters and therapy information. On the one hand, it will be necessary to know whether the program will allow patients to improve their lifestyles and maintain a good state of health, but on the other hand, it also has to evaluate how patients perceive the introduction of high-level technologies and its impact on their adherence to the program.

### Limitation

Contextual factors can influence the time of recruitment, which can affect the duration of data collection. Additionally, participant attrition may impact data analysis, even if considered when calculating sample size.

### Trial status

By the time the manuscript is submitted, the trial is recruiting patients. Current protocol version: 2.0 (19/11/2021). Recruitment of eligible subjects started in August 2022 and should end in September 2024.

### Supplementary Information

Additional file 1. SPIRIT 2013 Checklist.

## Data Availability

The study protocol submitted to the Ethics Committee is available from the lead investigators in the centers. Data collected in this protocol, completely unidentified, will be shared in an appropriate data archive.

## Abbreviations

HF: Heart failure
T2DM: Type 2 diabetes
IG: Intervention group
CG: Control group
CIRS: Cumulative Illness Rating Scale
BMI: Body mass index
HbA1c: Glycated hemoglobin
NT pro-BNP: Brain Natriuretic Peptide
6MWT: 6-Min walk test
PASE: Physical Activity Scale for the Elderly
MLHFQ: Minnesota Living with Heart Failure Questionnaire
DQOL: Diabetes Quality of Life
SF-12: Validated Italian version of the 12-item Short Form Survey questionnaire
SUS: System Usability Scale
